# Filter sterilized carcass rinsate for recovery of *Salmonella* species with various concentrations of cetylpyridinium chloride

**DOI:** 10.1002/fsn3.3463

**Published:** 2023-06-01

**Authors:** Douglas E. Cosby, Mark E. Berrang, Jonathan Frye, Arthur Hinton

**Affiliations:** ^1^ Poultry Microbiological Safety and Processing Research Unit U.S. National Poultry Research Center Athens Georgia USA; ^2^ Bacterial Epidemiology and Antimicrobial Resistance Research Unit U.S. National Poultry Research Center Athens Georgia USA

**Keywords:** antimicrobial interventions, carcasses, processing, *Salmonella*

## Abstract

Controlling *Salmonella* in poultry processing continues to be important to processors and consumers. Cetylpyridinium chloride (CPC) has proven to be effective in vitro in controlling *Salmonella.* This study evaluated the recovery of *Salmonella* after overnight storage in 4°C filter‐sterilized carcass rinsate containing CPC from 0.44 to 909 ppm (μg/mL). Ten *Salmonella* serotypes (18 strains), of which 6 serotypes are commonly isolated from poultry products, were grown in Bacto‐Tryptic Soy Broth overnight at 37°C. Serial dilutions of a CPC/propylene glycol solution were prepared in 24‐well tissue culture plates containing filter‐sterilized carcass rinsate. Approximately 10^7^ cfu/mL of each *Salmonella* serotype was added to the appropriate wells. Inoculated plates were stored overnight at 4°C. After storage, triplicate plates of brilliant green agar with sulfapyridine (BGS) were surface inoculated with 10 μL of the contents for each well, streaked for isolation, and incubated at 37°C for 24 h. Three replications were conducted. The presence of typical colonies on BGS plates was recorded as growth and verified through biochemical and serological testing. Of the serotypes chosen, *Salmonella* Kentucky, Dublin, and Enteritidis were the least resistant to CPC with a median minimum inhibitory concentration (MIC) of 14.22 μg/mL (range from 3.55 to 56.88 μg/mL); *S.* Typhimurium demonstrated a median MIC of 114.00 μg/mL (range from 28.44 to 114.00 μg/mL). Residual CPC potentially remaining attached to a carcass or in the weep after processing could potentially alter which *Salmonella* serotype is recovered from a carcass rinse due to different growth patterns during regulatory testing, with a potential for more virulent strains not to be recovered.

## Graphical abstract

Controlling *Salmonella* in poultry processing continues to be important to processors and consumers. This study evaluated the recovery of *Salmonella* after overnight storage in 4°C filter‐sterilized carcassS rinsate containing CPC from 0.44 to 909 ppm (μg/mL). *Salmonella* Kentucky, Dublin and Enteritidis were the least resistant to CPC and *S*. Typhimurium was the most resistant.

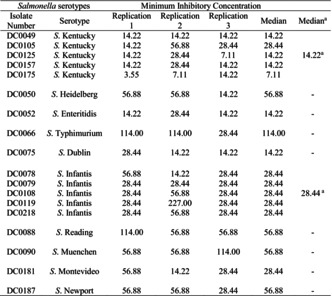

## 1 INTRODUCTION

*Salmonella enterica* species are gram‐negative rod‐shaped bacteria commonly found in the gastrointestinal tract of poultry (Brenner et al., 2000) and are a primary cause of foodborne illness in the United States. It is estimated that 938 million cases of salmonellosis occur every year globally with non‐typhoidal salmonellae as the cause (Antunes et al., 2016). Contaminated poultry products have been associated with approximately 25% of the outbreaks of foodborne salmonellosis (Akil & Ahmad, 2019). Contamination of poultry meat often occurs during processing from intestinal leakage (Berrang et al., 2001), contaminated processing equipment, water, and human contact. USDA‐FSIS data show the prevalence of *Salmonella* in raw carcasses and parts has remained constant. Between October 2018 and September 2019, the presence of *Salmonella* was 8.77% and 3.62% for parts and carcasses, respectively (Food Safety and Inspection Service, 2020).

On January 1, 2006 the European Union (EU, 2003) and on January 1, 2017, the United States Food and Drug Administration (FDA) banned the use of antibiotics in chicken feed as growth promoters (AccessScience, s.v, 2017). This ban has led to changes in the way antimicrobial/biocidal compounds are used in the processing plants in the United States (Hudson & Sukumaran, 2020). Greater emphasis has been placed on postharvest strategies to decrease the microbial presence in carcasses. Increased use of antimicrobial chemicals is one of the most common interventions in poultry processing currently (Kim et al., 2017). Commonly used processing aids includes cetylpyridinium chloride (CPC), peroxyacetic acid (PAA), sodium hypochlorite, and acidified sodium chlorite.

In a 2019 survey, Ebel et al. (2019), polled 167 U.S. poultry establishments which produce both carcasses and parts and determined plants used the following chemical interventions: PAA (74%), CPC (13%), acidified sodium chlorite (5.4%), sodium hypochlorite (1.8%), chlorine (0.6%), chlorine dioxide (0.6%), and other interventions (3.6%) were used for carcasses. For parts, establishments used CPC (4.2%), chlorine (1.8%), sodium hypochlorite (1.8%), acidified sodium chlorite (0.6%), other (3.0%), or no agents (1.2%). With increased regulations, more poultry processors are likely to increase the use of antimicrobials and/or biocides to reduce carcass and part contamination, especially if the plants are having difficulty meeting and maintaining the compliance standards.

Cetylpyridinium chloride is a water soluble, nonvolatile, quaternary ammonium compound (QAC) and has been found effective against various pathogenic bacteria without negatively affecting quality (Buncic & Sofos, 2012). Breen et al. (1995) reported that CPC (a quaternary ammonium compound) inhibited or reversed the attachment of *Salmonella* to chicken skin. Regulations require a water rinse and recapture of at least 99% of the antimicrobial for recycling ensuring that most of this antimicrobial is collected and not released into either the environment for small producers who may use collection ponds or into the local sewage/wastewater processing systems for larger producers who use municipal systems (Safe Foods Corporation, 2019).

It has been documented that QACs induce leakage of intracellular components which indicated membrane damage. CPC integrates into the lipid membrane of the bacterial cells which interferes with homeostasis and osmoregulation. The binding to anionic sites on the surface membranes of bacteria by CPC may cause slow or rapid cell death by damaging the cell walls resulting in leakage or solubilization of the cellular components depending on the concentration, low versus high (Morente et al., 2013).

Paul et al. (2017) reported that both serotype and level of organic matter contamination may significantly influence bacterial survival. It is important to note that most studies conducted in vitro to determine efficacy of biocides typically use one serotype without evaluating the impact of the presence of the antibacterial activity of the biocide being tested (Rhouma et al., 2021). The objective of this study was to evaluate the effect of one biocide, CPC, on the recovery of *Salmonella* serotypes in a conventionally used medium supplemented with unknown factors (fats, proteins, etc.) from carcasses in vitro.

## 2 MATERIALS AND METHODS

### 2.1 Carcass rinsate

Ten carcasses were obtained from a local processing plant and transported back to the U.S. National Poultry Research Center, Russell Research Center pilot processing plant on ice in individual plastic bags (CryoVac®, SealedAir). Carcasses were transferred to clean individual CryoVac® bags and 400 mL of buffered peptone water (BPW, Accumedia, Neogen Culture Media) was added before being rinsed using a mechanical shaker (Simmons Engineering Company, Inc.) for 60 s. The rinsate was pooled into one container before being filtered through a prefilter (Whatman #4, Whatman Ltd) and filter sterilized using 0.2 μm PES Nalgene bottle top filter (Nalgene, Thermo Scientific). The sterilized carcass rinsate was stored at −80°C until use.

### 2.2 Cetylpyridium chloride solution

A stock solution of CPC (2000 μg/mL, Safe Foods) was prepared in BPW with propylene glycol (3000 μg/mL, PG, Sigma Aldrich) to maintain CPC in solution and reduce absorption into treated poultry tissues (FDA, 2007). One milliliter of the filter‐sterilized carcass rinsate was placed into all the wells of the 24‐well tissue culture plate (Corning) and 1.0 mL of the CPC stock solution (2000 μg/mL of CPC/3000 μg/mL of PG) was placed into the first well (for an initial concentration of 1000 μg/mL CPC) and thoroughly mixed. Twelve wells were used per serotype with CPC concentrations from 909 to 0.44 μg/mL (Table 1) after adjusting the concentrations for the addition of the inoculum volume (0.1 mL per well). Mixing was conducted in the pipette tip used for transfer and 1.0 mL of the CPC/rinsate solution was discarded from the final well to maintain continuity in well volume.

**TABLE 1:** Dilution of chemicals (μg/mL) after addition of inoculum.

Cetylpyridinium chloride	Propylene glycol
909.00	1364.00
455.00	682.00
227.00	341.00
114.00	170.00
56.88	85.23
28.44	42.61
14.22	21.30
7.11	10.65
3.55	5.33
1.78	2.66
0.89	1.33
0.44	0.67

### 2.3 *Salmonella* isolates

Ten *Salmonella* serotypes (18 isolates, Table 2), six of which are commonly isolated from poultry and poultry products (Dr. Jonathan Frye, Bacterial Epidemiology and Antimicrobial Resistance Research Unit, U.S. NPRC), were selected for evaluation. All isolates were stored at −80°C in tryptic soy broth (TSB, Becton, Dickinson Co.) and 15% glycerol (Sigma‐Aldrich Chemicals) until resuscitated for use. Brilliant green agar with sulphapyridine (BGS, Neogen‐Accumedia) was used for the cultivation and recovery of all *Salmonella* serotypes. Inoculums of approximately 10^7^ colony‐forming units/mL (cfu/mL) were prepared by suspending each strain in sterile buffered peptone water (BPW, Neogen‐Accumedia) and adjusting the absorbance to 0.20 ± 0.02 at 540 nm using a Spectronic 20D+ spectrophotometer (ThermoSpectronics, Thermo Scientific). This provides approximately 10^8^ cfu/mL of each *Salmonella* strain which was followed by a 1:10 dilutions yielding approximately 10^7^ cfu/mL of each strain. The strains were stored on ice until used to inoculate individual wells of 24‐well tissue culture plates (Becton Dickinson Labware) which contained the diluted CPC/PG and filter‐sterilized carcass rinsate.

**TABLE 2:** *Salmonella* strains.

*Salmonella* serotypes	
Isolate number	Serotype
DC0049	*S*. Kentucky
DC0105	*S*. Kentucky
DC0125	*S*. Kentucky
DC0157	*S*. Kentucky
DC0175	*S*. Kentucky
DC0050	*S*. Heidelberg
DC0052	*S*. Enteritidis
DC0066	*S*. Typhimurium
DC0075	*S*. Dublin
DC0078	*S*. Infantis
DC0079	*S*. Infantis
DC0108	*S*. Infantis
DC0119	*S*. Infantis
DC0218	*S*. Infantis
DC0088	*S*. Reading
DC0090	*S*. Muenchen
DC0181	*S*. Montevideo
DC0187	*S*. Newport

### 2.4 Antimicrobial assay

The tissue plates were inoculated with 100 μL of the individual serotypes into each of 12 wells and stored overnight at 4°C. After overnight storage, 10 μL from each well was streaked for isolation onto triplicate BGS agar plates and incubated 24 h at 37°C. After incubation, plates were observed for growth. The first dilution without growth was determined to be the minimum inhibitory concentration. Colonies with typical morphology were stab inoculated into triple sugar iron (TSI, Becton, Dickinson Co.) and lysine iron (LIA, Becton, Dickinson Co.) agar slants for biochemical confirmation as *Salmonella*. Serological confirmation was completed using Difco *Salmonella* O‐antiserum (Becton, Dickinson Co.). Results are reported as minimum inhibitory concentration defined as the lowest concentration that will inhibit the growth of a microorganism after overnight incubation with confirmation on BGS plates without antibiotics and confirmed both biochemically and serologically. Three replicates were conducted, and the MIC concentrations are reported.

## 3 RESULTS AND DISCUSSION

*Salmonella* serotypes selected for this study were *S*. Kentucky (5 strains), Heidelberg, Enteritidis, Typhimurium, Dublin, Infantis (five strains), Reading, Muenchen, and Montevideo. *S*. Kentucky had a median MIC of 14.22 μg/mL across the five strains (range of 7.11–56.88 μg/mL). One strain each of *S*. Dublin and Enteritidis had the same median MIC of 14.22 μg/mL (14.22–28.44 μg/mL). *S*. Infantis had a median MIC of 28.44 μg/mL across five strains (14.22–227.00 μg/mL). *S*. Typhimurium had the highest median MIC at 114.00 μg/mL (28.44–114.00 μg/mL). The remaining four serotypes (*S*. Heidelberg, Muenchen, Newport and Reading) had a median MIC of 56.88 μg/mL (14.22–114.00 μg/mL; Table 3).

**TABLE 3:** Minimum inhibitory concentrations (μg/mL) of CPC in filter‐sterilized carcass rinsate.

*Salmonella* serotypes		Minimum inhibitory concentration				Median^a^
Isolate Number	Serotype	Replication 1	Replication 2	Replication 3	Median	
DC0049	*S*. Kentucky	14.22	14.22	14.22	14.22	14.22^a^
DC0105	*S*. Kentucky	14.22	56.88	28.44	28.44	
DC0125	*S*. Kentucky	14.22	28.44	7.11	14.22	
DC0157	*S*. Kentucky	14.22	28.44	14.22	14.22	
DC0175	*S*. Kentucky	3.55	7.11	14.22	7.11	
DC0050	*S*. Heidelberg	56.88	56.88	14.22	56.88	–
DC0052	*S*. Enteritidis	14.22	28.44	14.22	14.22	–
DC0066	*S*. Typhimurium	114.00	114.00	28.44	114.00	–
DC0075	*S*. Dublin	28.44	14.22	14.22	14.22	–
DC0078	*S*. Infantis	56.88	14.22	28.44	28.44	28.44^a^
DC0079	*S*. Infantis	28.44	28.44	28.44	28.44	
DC0108	*S*. Infantis	28.44	56.88	28.44	28.44	
DC0119	*S*. Infantis	28.44	227.00	28.44	28.44	
DC0218	*S*. Infantis	28.44	56.88	28.44	28.44	
DC0088	*S*. Reading	114.00	56.88	56.88	56.88	–
DC0090	*S*. Muenchen	56.88	56.88	114.00	56.88	–
DC0181	*S*. Montevideo	56.88	14.22	28.44	28.44	–
DC0187	*S*. Newport	56.88	56.88	28.44	56.88	–

^a^ Median MIC for five *S*. Kentucky and *S*. Infantis strains.

The additional strains of *S*. Kentucky and Infantis were selected because a previous study showed that these two serotypes were susceptible to the effect of CPC at very low concentrations (Cosby et al., 2018). In the present study, all 10 *Salmonella* serotypes exhibited growth at some level which contrasts with the data presented by Cosby et al. (2018) where *S*. Infantis (strain SQ0078) was inhibited by concentrations of 0.49 μg/mL across 6 replications of the study and the *S*. Kentucky (strain SQ0049) exhibited growth in only one of 6 replications. Several other serotypes exhibited no growth in various replicates during this study. The highest MIC exhibited during the previous study 0.98 μg/mL. In this study, we exhibit MIC's 20–85 times higher than the highest MIC in the previous study. An unknown factor present in the filter‐sterilized rinsate might be providing some protection for the *Salmonella* or organic material present in the rinsate might inactivate the antimicrobial.

Residual antimicrobials on the carcasses and parts as well as in the rinse medium could explain their perceived effectiveness, as growth inhibition, injury, and/or killing may continue in the packaged product due to the presence of residual chemicals. Gamble et al., 2016 determined that approximately 60, 26, and 2 mL of water (or dip solution) would be retained 0, 1, and 5 m after postchill dip treatments. This correlates to approximately 1200, 520, and 40 ppm (μg/mL), respectively, of CPC that might remain on carcasses and be incorporated into the carcass rinse and weep after a postchill antimicrobial treatment (most often a 20–30 s dip) after 0, 1, and 5 m of drip time. Additionally, after overnight storage, these researchers were unable to recover inoculated *Salmonella* into carcass rinses containing the appropriate concentration of CPC at 0 and 1 m post dip and only 5% of the *Salmonella* from the 5‐m postdip samples.

Mohammad et al. (2018) determined that neutralizing CPC with lecithin and polysorbate 80 in BPW (as used in Dey‐Engley (D/E) neutralizing buffered) allowed the recovery of *Salmonella* in vitro. Addition of these two neutralizers at 1X their respective content in D/E buffer was required to allow for the recovery of *Salmonella* isolates after being exposed to 0.6% (6000 μg/mL) and 0.8% (8000 μg/mL) CPC. Gamble et al. (2017) developed a neutralizing buffer capable of recovering *Salmonella* from carcass rinses where it was predicted that a final CPC concentration of 1200 ppm would be reasonably expected by incorporating 7 g/L lecithin into BPW. It was determined by these researchers that 2.6 molecules of lecithin were required to neutralize one molecule of CPC in the presence of 0.0236 mol/L of propylene glycol (a dispersing agent; Gamble et al., 2017).

These results indicate that the survival and recovery of *Salmonella* from carcass rinsates might be dependent on multiple factors. The residual antimicrobials might inhibit the recovery of foodborne pathogens in a regulatory laboratory by interfering with growth or killing the pathogens. Use of filtered sterilized rinsate is a fair approximation of the actual carcass rinse and was used to include the unknown factors present in carcass rinsate which might protect *Salmonella* serotypes from the bactericidal effect of the antimicrobials used currently in the industry. Future studies are planned to determine what factors present in the carcass rinsate (i.e., fats, proteins, etc.) might positively or negatively affect the recovery of not only *Salmonella* species but other human enteropathogens commonly found in poultry and poultry products.

## 4 CONCLUSIONS

In this study the effect of a novel substrate combined with a commonly used biocide on the recovery of *Salmonella* serotypes commonly associated with poultry and poultry products was investigated. The *Salmonella* serotypes selected for this study demonstrated a higher MIC when cultured in the rinsate than when grown in sterile BPW in a previous study. The mechanism of this higher resistance was not investigated in this study but will be investigated in future studies. This study demonstrated that additional factors found in the filter‐sterilized rinsate might increase the ability of *Salmonella* serotypes to resist the action of commonly used biocides similar to the factors which would be present in a real‐world carcass rinsate. These unknown factors might not be present when susceptibility testing is conducted using common media in a regulatory laboratory. Further research is necessary and planned to identify these unknown factors in order to improve the use of biocides as an intervention strategy for producers.

## AUTHOR CONTRIBUTIONS

**Douglas E. Cosby:** Conceptualization (lead); formal analysis (equal); investigation (equal); methodology (equal); supervision (equal); writing – original draft (lead). **Mark E. Berrang:** Conceptualization (equal); formal analysis (equal); methodology (equal); writing – review and editing (equal). **Jonathan Frye:** Conceptualization (equal); formal analysis (equal); methodology (equal); writing – review and editing (equal). **Arthur Hinton:** Conceptualization (equal); project administration (equal); resources (equal); supervision (equal); writing – review and editing (equal).

## CONFLICT OF INTEREST

None of the authors present a conflict of interest.

## ETHICS STATEMENT

No human subjects or animals were utilized in this study.

## Data Availability

Data will be available upon request from the Corresponding author.
